# Editorial Notes

**Published:** 1901-09

**Authors:** 


### Editorial Notes.

We have noticed elsewhere the irreparable loss sustained by the medical profession in the West of England by the very sudden and wholly unexpected death of Dr. Aust Lawrence.

Within the past few weeks two epoch-marking events have occurred in the medical world, the one of international importance, viz., the Tuberculosis Congress; the other involving the welfare of the British Medical Association, and likewise, therefore, though in a more restricted sense, of world-wide interest.

**British Congress on Tuberculosis.**—It is generally difficult to appreciate the historical importance of events until after the lapse of a considerable period; but if the united efforts of the whole civilised world to cope with the great plague of the twentieth century should in due course be followed by that large measure of success which there is a fair reason to anticipate, this British Congress will stand out conspicuously in the history of the country. Under the patronage of His Majesty the King, the head of the greatest empire the world has ever known, with Field-Marshal H.R.H. the Duke of Cambridge as President, officered by most distinguished politicians, as well as physicians and surgeons, with most of the highest experts on the subject throughout the world assembled together, the recent Congress marks the world's appreciation of scientific research, and the desire of the leaders in the political world for hearty co-operation with the medical profession, which is trusted to point the way to deliverance.

It is devoutly to be wished that science will prove triumphant, but we cannot disguise the fact that our knowledge is still very imperfect and that much yet remains for patient investigation. This, indeed, was brought home to everyone present by the unexpected announcement by Prof. Koch that he felt justified "in maintaining that human tuberculosis differs from bovine and cannot be transmitted to cattle," and that "whether man is susceptible to bovine tuberculosis at all is not yet absolutely decided . . . if such susceptibility really exists, the infection of human beings is but a very rare occurrence."

It is not the place here to discuss these vital questions, but however strong our convictions, up to the present, may have been adverse to Professor Koch's newly-expounded doctrine, we ought to keep an open mind and to remember that even his original statement that the *bacillus tuberculosis* was the essential cause of tuberculosis was received with difficulty by many of those who now have no doubts on that point. What strikes one at the outset is the weakness of the evidence he adduced in the course of his address in support of the startling upheaval of present-day convictions on the identity of "human tuberculosis" and bovine "perlsucht." We are glad, however, that King Edward, like the Government of Germany, has appointed a Commission to enquire into this important question, and we trust that it will not be long before the Royal Commission will be able to solve the problem.

Before we leave this question of tuberculous infection, we may remind our colleagues that Prof. Koch is by no means the first to take a firm stand against the identity of human and bovine tuberculosis. Indeed, as Dr. Latham points out,[1] as long ago as 1880 Virchow (although we believe he is now opposed to Koch's dictum) drew attention to the specific characters which distinguish human from bovine tuberculosis (perlsucht), stating that up to that time no individual has ever been known to contract pearl nodules from eating the flesh of animals affected with bovine tuberculosis. Furthermore, in the number of this *Journal* for March, 1899, Dr. Theodore Fisher gave an able summary of recent literature bearing on the question, and cited the observations of Carr, Colman, Kossel, Weiderhofer, Emmet Holt and Northrup, and also gave the results of his own experience in the *post-mortem* room of the Bristol Royal Infirmary, all affording strong presumptive evidence in favour of the view now publicly adopted by Koch. Dr. Fisher's own views may be gathered by the following sentences from his

[1] *Lancet*, 1901, ii. 415.

article: "Tuberculous disease in cows may be the same disease as in man, but it does not follow that the tubercle bacilli present in the milk of diseased animals are very virulent towards man. . . . I am not aware that there is yet proof that the tuberculous disease of cows is capable even of communicating a mild form of tuberculosis to man." We observe, too, that Adami now claims to have anticipated Koch in his conclusion. It is obvious that there is nothing new in Koch's dictum.

We cannot close our remarks on the Congress without noting the offer of Dr. Garnault to subject himself to inoculations with bovine tuberculosis, under Prof. Koch's supervision, and we sincerely hope that he may escape the penalty of his devotion to science. At the same time, we are decidedly opposed to such offers being accepted. We should be interested to know what attitude the anti-vivisectionists will take in regard to such experiments.

**British Medical Association Meeting at Cheltenham.**—Anything which concerns the essential interests of the British Medical Association affects the greater part of the British medical profession throughout the world; hence the very radical alteration in the constitution and government of the Association merits the most careful consideration. Hitherto the various branches have been represented by the members they elected to the Council, and the Council, though it has been stigmatised as an oligarchy, was nevertheless a representative governing body. Now, however, all members of the Association are to be attached to the branch in their respective districts, and every two hundred members are to elect a delegate to attend the annual meetings and to give expression to the wishes of the members they represent, the Council becoming neither more nor less than an executive committee to give effect to the resolutions of the newly-constituted democratic medical parliament. In theory this is an excellent arrangement, but whether it is a practical method of representing the wishes and intentions of the members as a whole is open to very grave doubt. Such an arrangement throws on each member the responsibility of keeping thoroughly in touch with all medical politics, and it is evident that unless busy medical practitioners are prepared to devote a good deal of thought and attention to the numerous questions that arise from time to time, they cannot form sound convictions enabling them to properly instruct their chosen delegate. To us it seems that a much better plan would have been to increase the members of the Council and thus have a more largely representative body of practitioners meeting at intervals throughout the year, interchanging views and forming opinions based on broader and fuller consideration of the best interests of the profession. On the newly adopted lines there will always be a great risk of small cliques carrying snap votes from which the delegate cannot escape and compelling his support to resolutions from which he may himself strongly dissent. That such dangers are not altogether hypothetical is plain from the very small numbers attending the meetings at Cheltenham, which carried in a somewhat arbitrary manner "the new constitution," meetings ostensibly called "to consider the report of the constitution committee."

The many charms of Cheltenham seem to have afforded a strong counter-attraction to medical politics, and undoubtedly the meeting there proved to be one of the most successful from the social as well as from the scientific standpoint that have been held for many years. One could scarcely fail to wonder why the fame of Cheltenham as a Spa should have lapsed. No one could attend the Mayor's reception at the Pittville Gardens without in some measure realising that the splendid pump room, like a huge Athenian temple of Hygeia, standing in its fine park, bespoke a glorious past; while the magnificent Winter Gardens, the Montpelier Gardens, the old Baths at Cambray, and the many beautiful boulevards seemed to us to render this town eminently well adapted for a modern health resort, not only analogous to, but in every sense equal to Homburg and many other Continental spas. [Dr. Luff read a paper in the Medical Section on the Waters of the Cheltenham Springs, and showed that it was as useful as those of many of the famous spas abroad, and there can be little doubt that if the town would spend a few thousand pounds for providing the baths and other appliances now considered essential to a thoroughly well equipped spa, it would not only be doing good to a large number of afflicted persons, but would repay itself many fold by enormously increasing local trade and the value of property.

But conflicting interests resulted lately in the rejection of a bill for this purpose in Cheltenham, and we fear that town will have to suffer much as Clifton is doing, from the inability of the more enterprising and far-seeing inhabitants to stir their fellow-citizens into a due appreciation of the latent wealth which lies at their doors; and meanwhile our German cousins will continue to flourish and grow fat on the efflux of our wealthy patients, who not unnaturally seek pleasurable roads to health where an effort is made to provide for their comforts and necessities.

M. Doyen's cinematographic exhibition of surgical operations, in the theatre at Cheltenham, should never be repeated at any medical gathering. The pictures serve no useful educational purpose whatever, and the dignity of the medical profession is certainly not enhanced by such displays. We question if they would be allowed in any representative medical gathering in France itself, and are surprised that the British Medical Association should have gone out of its way to afford Doyen opportunities for such ill-timed exhibitions at more than one of its annual meetings.

**Life Insurance.**—Medical men generally are being constantly called upon to act as medical advisers to one or another of the seventy-seven British offices enumerated in the index of the *Medical Annual* (page 717), as well as to a much larger number of foreign offices. This duty has often to be done with no previous training and with little knowledge as to what the requirements of the office may be. A series of very useful articles on the medical aspects of life insurance was published in the *Practitioner* of February last, and are well worthy of the careful study of all who are not familiar with the business of insurance offices.

The special functions of the medical examiner are very judiciously described by Dr. T. Colcott Fox, who states that "the faithful and adequate discharge of reporting on the value of the life of an applicant for insurance demands not only the application of a ripe medical knowledge, but a business aptitude, much tact, a gracious bearing, serenity of temper, the art of eliciting the essential information from every conceivable type of applicant, and particularly a clear conception of the task he is undertaking." Again, Dr. J. J. Perkins further points out that "of all his duties the prognosis of his cases has always been recognised as making the heaviest claim on the physician, and as needing the nicest application of his professional skill"; . . . and "after he has solved the question of whether to accept the life or not, he still has to face the further difficulty in those impaired lives of the penalty he should impose. Justice to the applicant and the interests of the office, in face of business competition, alike demand that the penalties imposed shall be adjusted as exactly as possible." This is just where the crux of the situation lies, for it is impossible that the ordinary medical examiner shall perform this duty in a just and equitable way unless he happens to be an insurance expert. How few are the examiners who study the "Proceedings of Life Assurance Medical Officers' Association," and who are qualified to do more than report on the case as to the facts which have been observed. In the opinion of Mr. James Chisholm, who sums up the question from the Insurance Companies' point of view, the officers often mislead the examiner by pushing their enquiries too far when they ask "how many years should be added to the age." We hold that this is a question for the insurance expert to determine, and that the medical examiner, away from the head office and not in touch with the actuary and directors, "is not competent, from the information within his reach, and from his want of knowledge of the conditions of the proposed contract, to form a fair and just opinion of what those terms should be." We hold that every client has a right to the benefits of insurance unless some good reason can be given why the office should decline to accept the risk; but it is for the office to determine, on the report of the medical examiner and all other evidence supplied, whether the risk should be accepted on ordinary terms or in what way those terms may be modified.

As regards the vexed question of fee, it appears to us that no office should accept anything but a complete examination, and no examiner can conscientiously report on any case in which the examination is not complete; the minimum fee of one guinea should always be paid, whether the sum to be insured is large or small. The examination must be the same, whatever the proposed sum may be, and there is no more reason for the proposal of a higher fee for the higher risks than there is for a reduction of the fee for the lower ones.

The Editorial staff of the *Journal* have much pleasure in offering their congratulations to Mr. J. Paul Bush, on the fact that his civilian services to the nation have been well recognised, and that his work in South Africa has won him an honourable distinction. It will be remembered that Mr. Paul Bush was invested by His Majesty King Edward VII., at Buckingham Palace, on June 3rd, with the order of the Companion of St. Michael and St. George, and he received his war medal on the Horse Guards' Parade, on June 12th, 1901. The members of the Bristol Medico-Chirurgical Society cannot do otherwise than feel that this distinction and dignity obtained by their Honorary Secretary reflects some honour on themselves.

